# Novel Nano-Liposome Formulation for Dry Eyes with Components Similar to the Preocular Tear Film

**DOI:** 10.3390/polym10040425

**Published:** 2018-04-11

**Authors:** Marta Vicario-de-la-Torre, María Caballo-González, Eva Vico, Laura Morales-Fernández, Pedro Arriola-Villalobos, Beatriz de las Heras, José Manuel Benítez-del-Castillo, Manuel Guzmán, Thomas Millar, Rocío Herrero-Vanrell, Irene T. Molina-Martínez

**Affiliations:** 1 Pharmaceutical Innovation in Ophthalmology Research Group, UCM 920415, Department of Pharmaceutics and Food Technology, Faculty of Pharmacy, Complutense University of Madrid, Plaza Ramón y Cajal, s/n, 28040 Madrid, Spain; martavicario@nutraessential.com (M.V.-d.-l.-T.); mcaballo@farm.ucm.es (M.C.-G.); rociohv@farm.ucm.es (R.H.-V.); 2 Ocular Surface and Inflammation, Ophthalmology Department, San Carlos Clinical Hospital, Calle Profesor Martín Lagos, s/n, 28040 Madrid, Spain; ebusqui@gmail.com (E.V.); lauramoralesfernandez@gmail.com (L.M.-F.); pedro_arriola@hotmail.com (P.A.-V.); benitezcastillo@gmail.com (J.M.B.-d.-C.); 3 Sanitary Research Institute of the San Carlos Clinical Hospital (IdISSC) and the Ocular Pathology National Net (OFTARED) of the Institute of Health Carlos III, Calle Profesor Martín Lagos, s/n, 28040 Madrid, Spain; lasheras@farm.ucm.es; 4 Department of Pharmacology, Faculty of Pharmacy, Complutense University of Madrid, Plaza Ramón y Cajal, s/n, 28040 Madrid, Spain; 5 Department of Biomedical Sciences, Faculty of Pharmacy, University of Alcalá, Ctra Madrid-Barcelona, Km 33.6, 28801 Alcalá de Henares, Madrid, Spain; manuel.guzman@uah.es; 6 School of Science and Health, Western Sydney University, Rydalmere Sydney, New South Wales 2116, Australia; t.millar@westernsydney.edu.au; 7 Instituto Universitario de Farmacia Industrial (IUFI), Faculty of Pharmacy, Complutense University, 28040 Madrid, Spain

**Keywords:** artificial tears, preocular tear film, tear break-up time, liposomes, mucoadhesive polymers

## Abstract

Dry eye is commonly treated with artificial tears; however, developing artificial tears similar to natural tears is difficult due to the complex nature of tears. We characterized and evaluated a novel artificial tear formulation with components similar to the lipid and aqueous constituents of natural tears. Nano-liposomes, composed in part of phosphatidylcholine, were dispersed in an aqueous solution of bioadhesive sodium hyaluronate. Liposome size, zeta potential, and physicochemical properties of the fresh and stored (4 °C) liposomal formulation were analyzed. In vitro tolerance was tested using human corneal and conjunctival cell lines by exposures of 15 min to 4 h. The tolerance of the liposomal formulation was evaluated in animals (rabbits). The average liposome size was 186.3 ± 7.0 nm, and the zeta potential was negative. The osmolarity of the formulation was 198.6 ± 1.7 mOsm, with a surface tension of 36.5 ± 0.4 mN/m and viscosity of 3.05 ± 0.02 mPa·s. Viability values in the human corneal and conjunctival cell lines were always >80%, even after liposomal formulation storage for 8 weeks. Discomfort and clinical signs after instillation in rabbit eyes were absent. The new formulation, based on phosphatidylcholine-liposomes dispersed in sodium hyaluronate has suitable components and characteristics, including high in vitro cell viability and good in vivo tolerance, to serve as a tear substitute.

## 1. Introduction

**Figure 1:**
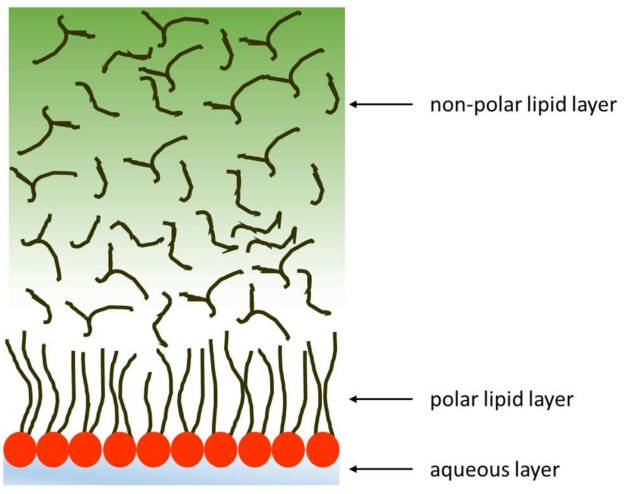
Schematic structure of the tear film.

Dry eye disease (DED) is a multifactorial syndrome involving alterations of the preocular tear film and ocular surface tissues that result in symptoms such as discomfort, visual disturbance, tear film instability, and inflammation [1,2]. The preocular tear film is a complex and dynamic structure formed by lipid and aqueous components (Figure 1). The aqueous phase is a hydrated gel made up of proteins, mucins, and water-soluble substances such as electrolytes, sugars, growth factors, and vitamins [3]. The aqueous phase is covered by a lipid layer that facilitates spreading and stability of the preocular tear film [4,5], and it resists collapse of the film [6]. The lipid phase is composed of two fractions: an outer non-polar one at the air interface and an inner polar component in contact with the aqueous-mucin layer. The non-polar hydrophobic lipids are cholesterol esters, wax esters and triglycerides. In tears, the polar amphiphilic lipids are mainly phospholipids, glycosphingolipids, and ceramides. Phospholipids comprise 5–15% of the total lipid fraction, and the main component, phosphatidylcholine, is close to 40% of the total polar lipids [7,8,9,10,11].

Artificial tears are the preferred first-line therapy for the treatment of DED because they relieve the symptoms of the disease and contribute to the hydration, lubrication, stability, and optical properties of the ocular surface with low side effects. Artificial tears are often formulated with mucoadhesive polymers such as cellulose derivatives or sodium hyaluronate (SH) that increase the residence time of the formulation and safeguard the ocular surface. Recent advances in the understanding of the pathogenesis of DED have led to changes in the therapeutic management such as low tear osmolarity and addition of osmoprotectants or oily substances [12,13]. Nevertheless, in severe grades of the disease, a more complex therapy with an anti-inflammatory basis is required [14].

Even though there are many commercial artificial tears [15], none are similar in composition to natural tears, which are extremely complex. Each component of the preocular tear film has a unique physico-chemical property that plays a role in the homeostatic maintenance of the ocular surface [16].

In the 1970s, Holly and Lemp showed that for a thin film of water to spread over a surface, physical chemistry requires that the adhesiveness of the surface must be relatively high and the surface tension of the aqueous component relatively low [17]. Based on this underlying principle, we have developed a novel artificial tear formulation that includes SH, a component similar to the tear film mucin that increases adhesion to the ocular surface. The formulation also includes liposomes made from phospholipids that are similar to the composition of the lipid layer and that decrease surface tension. The liposomes also contain vitamin E. Once prepared, the liposomes are dispersed in a hypotonic aqueous solution of SH and trehalose. In this report, we investigate the in vitro and in vivo properties of this formulation.

## 2. Materials and Methods 

### 2.1. Reagents

Phospholipon 90G containing >95% phosphatidylcholine (PC) purified from soy lecithin was purchased from Phospholipid GmbH (Cologne, Germany). For Langmuir trough experiments, cholesterol (Ch), α-tocopherol (vitamin E), benzalkonium chloride (BAK), boric acid, 3(4,5-dimethylthiazol-2-yl)-2,5-diphenyltetrazolium bromide (MTT), disodium borate, and other standard salts or chemicals were acquired from Sigma-Aldrich Chemical Co., Madrid, Spain, or from Sigma-Aldrich, Sydney, Australia. Trehalose was purchased from Cymit Química S.L. (Barcelona, Spain), and SH ophthalmic grade (*M*_w_ 400,000–800,000 g/mol) from Abarán Materias Primas (Madrid, Spain). Cell culture reagents were purchased from Gibco-Invitrogen (Life Technologies, Barcelona, Spain). Male New Zealand white rabbits, 2.0–2.5 kg, were purchased from San Bernardo Farm (Navarra, Spain).

### 2.2. Preparation of Liposomal Formulation

Liposomes were prepared according to the technique described by Bangham et al. [18] and modified by our research group [19]. Briefly, PC, Ch, and vitamin E (8:1:0.08; mg/mL) were dissolved in chloroform, and the organic solvent was slowly removed using a rotavapor (Büchi^®^, Massó-Analítica, Barcelona, Spain) at 33 °C. The thin film of dry lipids that formed on the inner surface of the flask was hydrated with an aqueous solution of trehalose (1.6%) and 135.5 mM borate-buffered solution. This initial liposomal dispersion was designated as F0 (Table 1). Trehalose, a cryoprotective sugar, was employed to adjust the osmolarity to hypotonic values and to improve survival of cultured cells exposed to desiccation-induced stress [20,21].

A second liposomal dispersion was formed by 10 cycles of F0 extrusion through polycarbonate membranes (0.22-µm pore size) (Sartorius, Göttingen, Germany) by a high-pressure extruder (Lipex Biomembrane^TM^, Vancouver, BC, Canada). These nano-liposomes were left overnight at 4 °C to allow full hydration. To prepare the final formulation, the liposome dispersion was then diluted 1:2 with 0.4% SH in an aqueous solution of 135.5 mM borate and 42.3 mM trehalose. The final liposomal formulation, designated as FLF, was obtained after filtration through 0.22-µm pore membrane (Table 1). The concentration of liposomes, measured as the concentration of PC in the FLF, was 10 mg/mL. PC concentration was measured by colorimetric titration [19].

To ensure sterility, preparation of the ophthalmic formulation was done under aseptic conditions. All glassware was sterilized by dry heat and the aqueous solutions were sterilized by autoclaving. 

### 2.3. Characterization of the Final Liposomal Formulation 

#### 2.3.1. Mean Particle Size and Zeta Potential

Particle size and zeta potential were determined by photon correlation spectroscopy (Zetatrac^®^: Microtrac-Europe GmbH, Meerbusch, Germany). Studies were performed in triplicate on freshly prepared FLF diluted 1:20 (*v*/*v*) in MilliQ^®^ water at room temperature (25 °C). Additional stability studies were performed following the same protocol after 1, 2, 4, and 8 weeks of storage at 4 °C in the dark. 

#### 2.3.2. pH, Osmolarity, Surface Tension, Dynamic Surface Pressure, and Viscosity

The pH of the FLF was determined using a pH meter (model 230, Mettler Toledo, Barcelona, Spain) calibrated at pH 7.0 and pH 9.0 with standard solutions (Crison, Alella, Barcelona, Spain). Measurements were performed in triplicate at room temperature. 

The osmolarity was analyzed by vapor pressure measurements with an osmometer (K-7000, Knauer, Berlin, Germany) after calibration with a 400 mOsm NaCl solution. Measurements were performed in triplicate at 33 °C, which corresponds to ocular surface temperature [22]. 

The FLF viscosity was assessed with a thermostatically controlled rheometer (Rheostress R1, Haake, Düsseldorf, Germany) using a parallel plate geometry (60-cm diameter, 0.5-mm gap). High shear rates were applied [23]. Viscosity was measured when the steady state was reached with shear rates increasing from 0 to 1000 s^−1^. All determinations were made in triplicate at 33 °C.

Surface tension of the FLF was measured with a tensiometer (K-11, Kruss, Hamburg, Germany) by the Wilhelmy plate method in triplicate at 33 °C. Prior to testing the samples, the tensiometer was calibrated using MilliQ^®^ water.

Dynamic surface pressure measurements were carried out at 20 °C on an 80-cm^2^ Langmuir trough with double barriers (NIMA 102M; Nima Technology Ltd., Coventry, UK) using techniques described previously [24]. The FLF was initially diluted 1:100 with water and then 20, 50, or 100 µL were applied to the surface of the artificial tear buffer subphase between the barriers. Twenty minutes were allowed for equilibration, and then compression and expansion isocycles at 15 cm^2^·min^−1^ were carried out. 

Stability of the FLF pH, osmolarity, viscosity, and surface tension was also determined after 2, 4, and 8 weeks of storage at 4 °C in the dark. 

### 2.4. In Vitro Tolerance Studies

In vitro tolerance of freshly prepared FLF and of FLF stored for 2, 4, and 8 weeks at 4 °C in the dark was evaluated with two different ocular cell lines: (1) immortalized human corneal-limbal epithelial (HCLE) cells generously donated by Dr. Ilene K. Gipson (Schepens Eye Research Institute, Harvard Medical School, Boston, MA, USA) [25] and (2) normal human conjunctiva (IOBA-NHC) cells [26] generously donated by the Institute for Applied Ophthalmobiology (University of Valladolid, Valladolid, Spain). HCLE cells were cultured in a keratinocyte serum-free medium (SFM), supplemented with 0.5 mL CaCl_2_ 0.3 M, 1.25 mL bovine pituitary extract, and 40 µL epidermal growth factor (EGF). IOBA-NHC cells were cultured in DMEM/F12 medium supplemented with 10% calf serum, 2% penicillin-streptomycin, 2.5 µg/mL amphotericin B, 1 µg/mL bovine pancreas insulin, 0.5 µg/mL hydrocortisone, 0.1 µg/mL cholera toxin, and 0.2 ng/mL EGF.

For cytotoxicity assays, HCLE cells and IOBA cells were seeded into 96-well culture plates (40,000 cells/well) and cultured at 37 °C for 24 h in a 5% CO_2_ atmosphere [27]. Adherent cells were exposed to FLF for 15 min (short-term), 1 h (intermediate-term), or 4 h (long-term). These times were chosen to emulate the types of dwell times on the ocular surface of various formulations [28]. An isotonic NaCl solution with 0.005% BAK was used as a positive control [29].

Cell viability was determined by the MTT assay, a measure of the redox activity of living cells [30,31]. The cells were incubated in a MTT solution (5 mg/mL in phosphate-buffered saline) for 3 h at 37 °C. After careful aspiration of the MTT solution, the cells were solubilized with dimethyl sulfoxide (100 µL/well). The extent of the reduction of MTT to formazan by the cells was quantified by optical density measurements at 550 nm using a plate reader (Digiscan mod 6010152EU, Eugendorf, Austria). Viability was set as 100% for untreated cells. Eight wells per sample were tested, and the assays were performed in triplicate. The results were expressed as the reduction in cellular viability compared to negative controls for at least three independent experiments. 

### 2.5. Desiccation Assay

The same vehicle solutions in which the liposomes were dispersed, i.e., either 0.2% SH, 135.5 mM borate, and 42.3 mM trehalose or NaCl with 0.2% SH, were tested for the ability to prevent desiccation in IOBA-NHC cells. For the desiccation assays, neither vehicle contained dispersed liposomes. 

The protocol employed was based on previously reported desiccation assays [32,33,34]. Cells were seeded at a density of 40,000 cells/well. On confluent cell growth, cells were incubated (1 h) with 100 µL of either the trehalose-borate-SH solution or the NaCl-SH solution. After incubation, the vehicles were discarded, and cells were exposed to a constant-flow fume hood without any liquid for 0 and 15 min. To assess viability, the cells were incubated with the MTT reagent (5 mg/mL) for 3 h at 37 °C, as previously described. Cell viability was set up at 100% for undesiccated cells. 

### 2.6. Animals

Male white New Zealand rabbits, housed in individual cages in the Animal Laboratory of the Complutense University (Madrid, Spain), had free access to food and water. They were acclimated for two weeks in rooms maintained at 22 °C and 50% relative humidity with controlled 12/12 h light/dark cycles. All of the protocols herein were approved by the Ethics Committee for Animal Research of Complutense University of Madrid. All animal manipulations followed institutional guidelines, European Union regulations for the use of animals in research (European Communities Council Directive (86/609/EEC), and the ARVO (Association for Research in Vision and Ophthalmology) statement for the use of animals in ophthalmic and vision research.

### 2.7. Tolerance Assays in Rabbits

In vivo tolerance of the FLF was evaluated following a protocol previously described [35] and based on the test guidelines of Organization for Economic Co-Operation and Development (Table 2). The right eye of each rabbit (*n* = 6) received 30 µL of the FLF (treatment group), and the contralateral eye received topical administration (30 µL) of an isotonic solution of sodium chloride (control group) by using a micropippete. Both ophthalmic preparations were administered every 30 min for 6 h in the cul-de-sac of the rabbits’ eyes. The ocular surface status was analyzed immediately before the first application and at 3, 6, and 24 h after the first instillation of the preparations. Animal discomfort and signs and symptoms of the cornea, conjunctiva, and lids were carefully studied following the guidelines.

### 2.8. Statistical Analysis 

Data were expressed as means ± standard error of the means. Statistical differences among mean values were analyzed by multivariate analysis of variance (ANOVA). When necessary, two-tailed Student’s t-test was employed. Graphical analyses were carried out with Origin^®^ Pro8 software (Originlab, Northampton, MA, USA). Results were considered statistically significant when the *p*-values were <0.05.

## 3. Results

### 3.1. Characterization of the Liposomal Formulation (LF)

#### 3.1.1. Mean Particle Size and Zeta Potential

**Figure 2:**
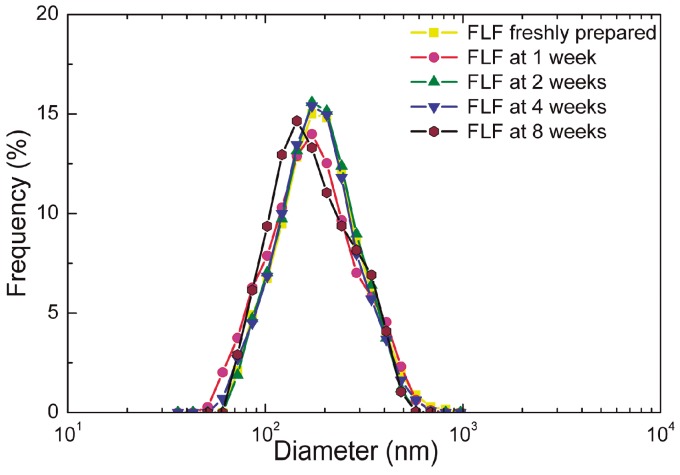
Size distribution of liposomes in the final liposome formulation (FLF). FLFs were stored for the period indicated at 4 °C in the dark. *n* = 3.

Photon correlation spectroscopy indicated that the nano-liposomes of the novel formulation had a unimodal size distribution with a diameter of 186.3 ± 7.0 nm (Figure 2, Table 3). The size distribution and diameter remained stable when stored up to eight weeks at 4 °C in the dark. The zeta potentials of the FLF were negative (from −13.3 mV ± 0.5 to −22.4 mV ± 1.6) in all cases and over time (Table 3) indicating stability of the dispersion and resistance to aggregation. Differences between baseline values and after different storage times were not significant. 

#### 3.1.2. pH, Osmolarity, Surface Tension, Viscosity, and Dynamic Surface Pressure

The pH, osmolarity, surface tension, and viscosity of the freshly prepared FLFs remained stable during the 8 weeks of storage (Table 4). Thus, storage for eight weeks had no effect on these FLFs physical parameters. 

**Figure 3:**
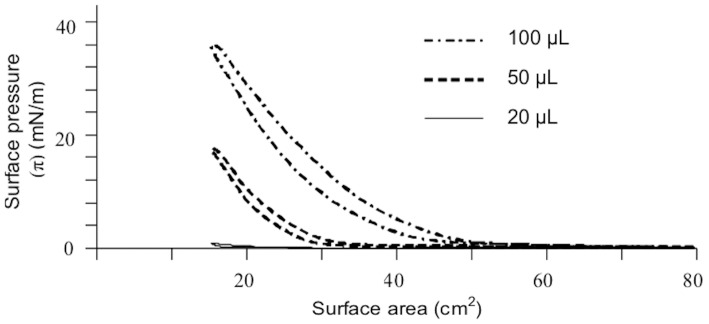
Pressure/area isocycles for different amounts of FLF diluted 1:100 in water and then applied to the surface of an artificial tear buffer.

Measurement of the surfactant properties of the FLF was performed using a Langmuir throw with moveable barriers that allows dynamic measurements with different concentrations of the dispersion. The liposomal formulation was compressed and expanded thanks to the movement of the barriers varying from a maximum of 80 cm^2^ to a minimum of 15 cm^2^. In these conditions the surface pressure was continuously monitored. Dynamic surface pressure measurements indicated that the FLF was surface active, and this activity depended on the concentration applied in the trough (Figure 3). The FLF was diluted 100-fold before being added to the trough, and the maximum surface pressures indicated a high surface activity.

### 3.2. In Vitro Tolerance Studies

**Figure 4:**
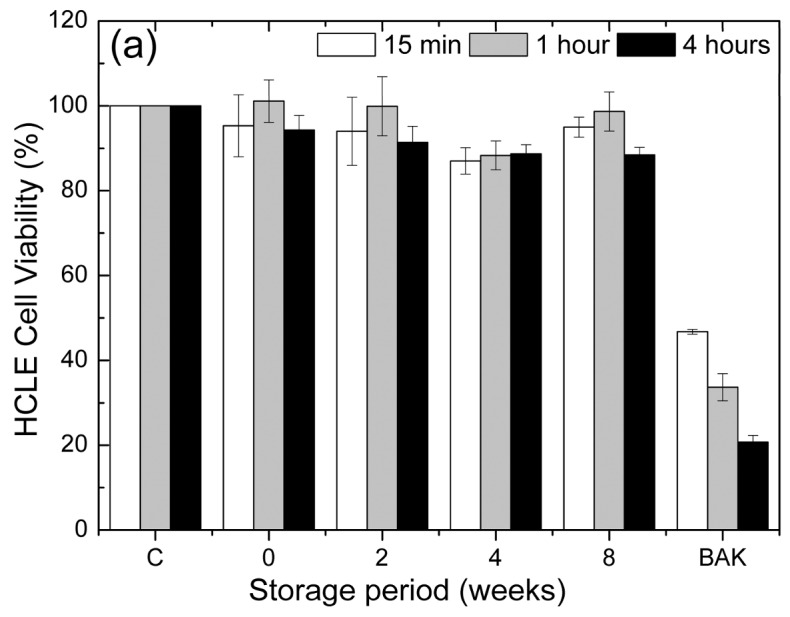

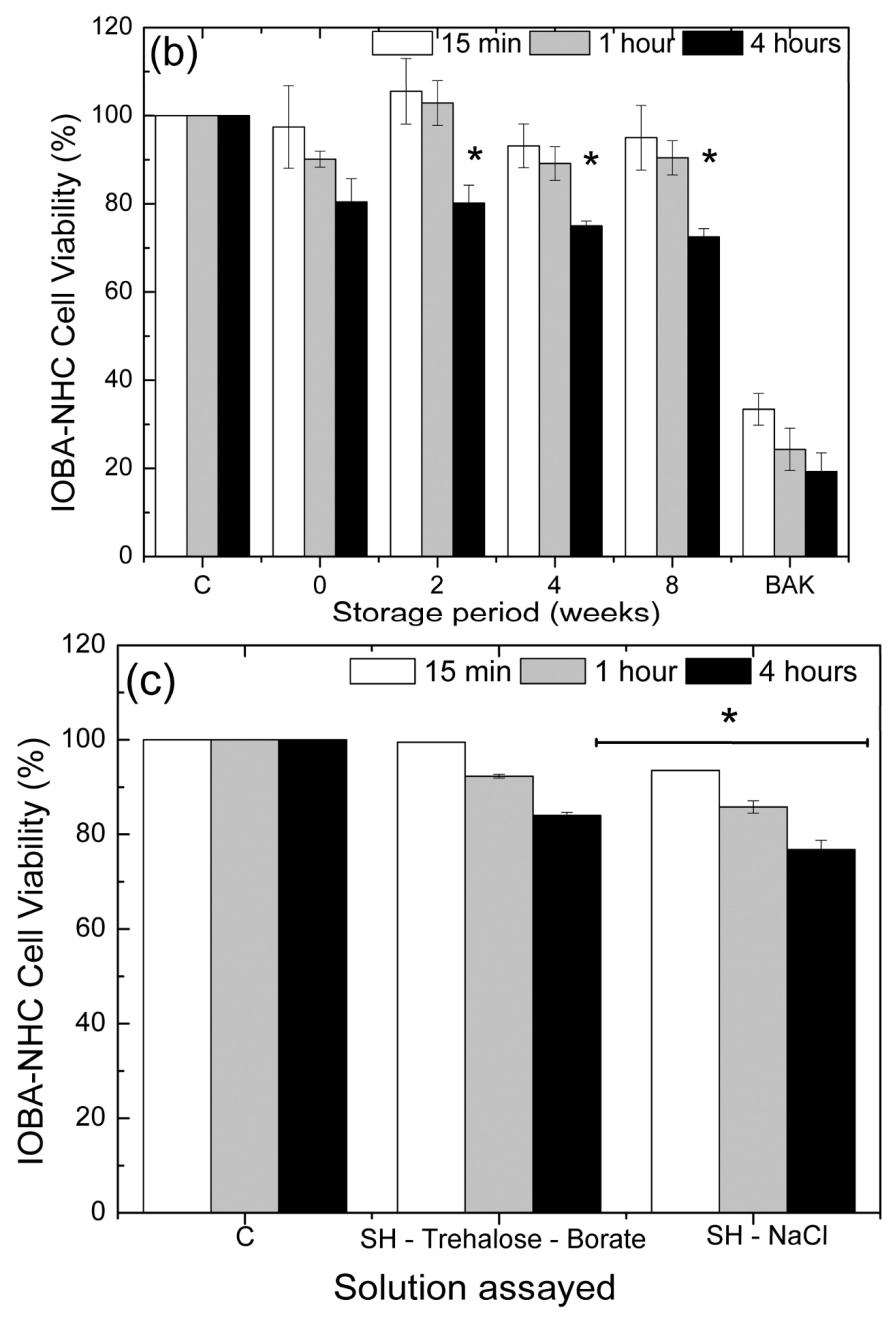
Viability of human corneal-limbal epithelial (HCLE) cells and Institute for Applied Ophthalmobiology normal human conjunctiva (IOBA-NHC) cells exposed to control and liposome formulations. The study was performed with freshly prepared FLFs and after storage for 2, 4, and 8 weeks at 4 °C in the dark. Exposure times were 15 min, 1 h, and 4 h. C, untreated cells were used as negative control; 0, FLF freshly prepared; 2, FLF stored for two weeks; 4, FLF stored for four weeks; 8, FLF stored for eight weeks; BAK, 0.005% benzalkonium chloride was used as the positive control. (**a**) For HCLE cells, there were no significant differences in viability among the different storage times and exposure times; (**b**) For IOBA-NHC cells, storage for 2, 4, and 8 weeks reduced the viability of cells exposed for four hours * *p* < 0.01 compared to the FLFs stored for the same period; (**c**) Cell viability of IOBA-NHC cells exposed to control and different solutions employed for liposomal dispersion. Exposure times were 15 min, 1 h, and 4 h. C, untreated cells were used as negative control; SH-trehalose-borate, a vehicle composed of sodium hyaluronate, trehalose, and borate-buffered solution used in the liposome dispersal in the present work; SH-NaCl, a vehicle composed of sodium hyaluronate (SH) and sodium chloride that was used to disperse liposomes in a previous work [19]. Neither vehicle in these experiments contained liposomes. Results were normalized to the viability of untreated cells. For C, the error bars were too small to be seen clearly. * *p* < 0.05 compared to untreated cells and to SH-trehalose-borate-treated cells at the same exposure time.

HCLE and IOBA-NHC cell viabilities were greater than 80% for up to 4 h of FLF exposure (Figure 4). After exposures of 15 min, 1 h, and 4 h to FLFs stored for different periods, HCLE cell viability values indicated that the FLFs were non-toxic for up to eight weeks in storage (Figure 3a). Similar viabilities were achieved when IOBA-NHC cells were exposed to FLFs for 15 min and 1 h, and there was no significant difference in the viabilities for these two times of exposure (*p* > 0.05, Figure 3b). Exposure of IOBA-NHC cells for 4 h to FLFs stored for two or more weeks resulted in viabilities that were lower than those for 15 min and 1 h (*p* < 0.01, Figure 3b). As expected, the BAK solution was toxic to cells, with IOBA-NHC cells being more sensitive than HCLE cells. 

The liposome vehicle, composed by trehalose, borate buffered solution, and sodium hyaluronate (SH), was also tested on the IOBA-NHC cells. For comparison, a previously described vehicle [19] containing SH and sodium chloride was also evaluated. This NaCl-SH vehicle had cell viability values lower than 80% at four hours of exposure while the vehicle containing trehalose, borate buffer solution, and SH exhibited 87.5% cell viability (*p* < 0.05, Figure 4c).

### 3.3. Desiccation Assay

**Figure 5:**
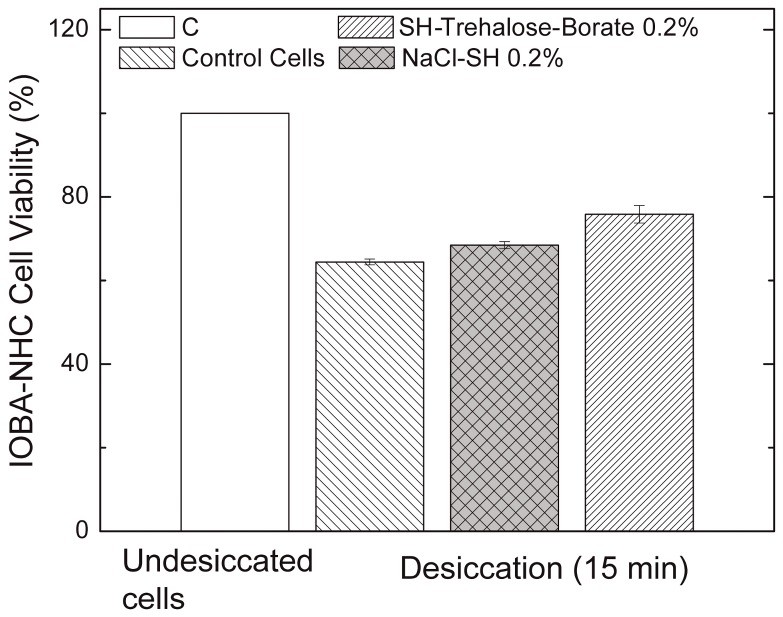
Viability of IOBA-NHC cells exposed to desiccation after incubation in trehalose-borate-boric acid buffer containing SH 0.2% and isotonic NaCl with SH 0.2%. Following 1 h of incubation with either trehalose-borate-boric acid buffer containing SH 0.2% or isotonic NaCl with SH 0.2% for 1 h, the IOBA-NHC cells were exposed to desiccation conditions for 15 min. Control cells were incubated in DMEM/F12 for 1 h and then desiccated as described for the experimental cells. Cell viability was measured by MTT assay. Error bars for the undesiccated cells are too small to be seen clearly. *n* = three experiments.

The viability of IOBA-NHC cells exposed to desiccation for 15 min was 64.4 ± 1.2% if left untreated. The viability of the desiccated cells treated with a vehicle containing sodium hyaluronate, SH, and sodium chloride, NaCl-SH, was 68.5x ± 1.4%, while those treated with trehalose-borate-boric acid-SH was 75.8 ± 3.6% (*p* < 0.03 vs. NaCl-SH; *p* < 0.006 vs. untreated control; Figure 5).

### 3.4. Tolerance Assays in Rabbits

None of the rabbits appeared to be in any discomfort before or immediately after the first FLF instillation or at the 3-, 6-, and 24-h examinations (Table 5). Further, there were no clinical signs of toxicity such as corneal opacity, hyperemia, or conjunctival and lid alterations at any point of the study. 

## 4. Discussion

These initial studies demonstrate that a formulation based on different components of the tear film is likely to be useful for the treatment of DED, a condition that affects 14–33% of the population [36]. Besides being non-toxic to the ocular surface, formulations need to achieve the pharmaceutical requirements described in the pharmacopoeia [37]. 

Regarding important technological properties, the FLF maintained consistency in liposome size, viscosity, and osmolarity for up to eight weeks of storage at 4 °C. Furthermore, there was no evidence of creaming over the test period (data not shown). One of the main drawbacks described for liposomes is poor stability in aqueous media [38]. In the present formulation, the liposome size remained stable with a unimodal size distribution for eight weeks when stored at 4 °C. This finding is probably due to the presence of the bioadhesive polymer SH in the aqueous solution. From a technological point of view, SH behaves as a protective colloid and prevents the creaming effect (data not show). The FLF showed a Newtonian behavior with a viscosity value of 3.05 ± 0.02 mPa·s, which is close to that of natural tears, 1–10 mPa·s [39], and this was unchanged over the duration of the storage period. The FLF was non-toxic and well-tolerated by the ocular surface cells in vitro and in vivo. Also, it did not show any adverse effects in rabbits. 

In the present study, the SH probably increased the surface adhesion to the cornea and the PC and Ch probably decreased the surface tension at the air-liquid interface. These two combined actions would help achieve the proper wettability of the ocular surface [40] and thus improve the stability of the tear film [17]. After instillation of the formulation, the liposomes could have either maintained their structure or reorganized according to their amphiphilic properties. In either case, a lipid layer on the ocular surface is likely to have formed. Further studies are necessary to determine the behavior of the liposomes after instillation.

It is generally accepted that trehalose protects cells from desiccation [41]. In the eye, this agent improves the epithelial damage in a reconstructed human corneal epithelium [42] and has an anti-inflammatory response in vitro. In a murine model of dry eye, application of trehalose eyedrops alleviated corneal damage and reduced conjunctival inflammation by decreasing the levels of inflammatory cytokines [20,21]. Trehalose-based formulations have significantly improved dry eye signs and symptoms compared to therapies based on hyaluronan or cellulose derivatives [43]. 

Compared to saline solution, trehalose in the current liposomal formulation increased the viability of IOBA-NHC cells. In a previous work, the liposomes were dispersed in SH and sodium chloride solution. This aqueous vehicle showed cell viability values lower than 80% at long term exposure (4 h) indicating poor tolerance [19]. However, in the current formulation, the vehicle included trehalose and borates with the SH, which increased the cell viability to 87.5%. Furthermore, trehalose is an effective osmoprotectant in the treatment of DED-associated hyperosmolarity [44]. According to its properties, trehalose is a key compound in the novel liposome formulation because of its protective effect on the stress caused by hyperosmolarity. In the current work, the use of a trehalose, borate-buffered solution and SH was superior in protecting conjunctival cells from desiccation with respect to the NaCl and SH solution. 

The novel liposome formulation also contained borate buffer because it exhibits antimicrobial activity [45]. Further, it does not induce calcium deposition in the cornea, which is an adverse effect associated with phosphate-buffered solutions [46]. SH was incorporated due to its bioadhesive properties that allow increased retention time on the ocular surface [27]. It also has wound healing activity and mucin-like behavior that provides improved lubricating action [47].

An important advantage of liposomes is that they can be loaded with active agents such as lipid- and/or aqueous-soluble drugs [48,49]. This feature suggests the possibility of promising improvements in chronic diseases in which the ocular surface suffers damage from the treatment itself. In fact, we recently used an anti-glaucoma liposome formulation that was enhanced with a hypotensive agent and bioadhesive polymers [50], but otherwise similar to the one reported here. The objective was to develop an effective hypotensive therapy for glaucoma that was not disruptive to the ocular surface, unlike other commercial eye drops that contain surface-damaging preservatives, especially in long-term treatments. 

The FLF developed in this work combines the beneficial effects of phosphatidylcholine liposomes, vitamin E, SH, and trehalose in a unique eye drop [51,52]. This novel artificial tear is designed as a tear replacement by adding components similar to those found in natural tears. As we found, the novel FLF provided beneficial effects on a human conjunctival cell line exposed to desiccation. Although the causes of DED are complex, loading active agents in this liposomal formulation could provide novel opportunities to enhance dry eye therapy.

## Figures and Tables

**Table 1:** Composition of the liposomal formulations.

Formulation	Composition
F0	20 mg/mL PC, 135.5 mM H_3_BO_3_, 2.0 mM Na_2_BO_4_, 42.3 mM trehalose
FLF	10 mg/mL PC, 135.5 mM H_3_BO_3_, 2.0 mM Na_2_BO_4_, 42.3 mM trehalose, 0.2% SH

F0: Initial formulation; FLF: Final liposomal formulation; PC: phosphatidylcholine, SH: sodium hyaluronate. The concentration of trehalose was adjusted to achieve an osmolarity value within an acceptable range (150–320 mOsm/L) for ophthalmic administration.

**Table 2:** In vivo tolerance grading system for macroscopically evaluated signs.

Grade	Discomfort	Cornea	Conjunctiva	Discharge	Lids
0	No reaction	No alterations	No alterations	No discharge	No swelling
1	Blinking	Mild opacity	Mild hyperemia/mild edema	Mild discharge without moistened hair	Mild swelling
2	Enhanced blinking/intense tearing/vocalizations	Intense opacity	Intense hyperemia/intense edema/hemorrhage	Intense discharge with moistened hair	Obvious swelling

**Table 3:** FLF diameter and zeta potential.

Storage Period (Weeks)	Size (nm)	Zeta Potential (mV)
0	186 ± 7	−21.8 ± 2.9
1	185 ± 6	−16.4 ± 1.5
2	191 ± 1	−13.3 ± 0.5
4	187 ± 3	−19.4 ± 0.7
8	181 ± 6	−22.4 ± 1.6

FLF: Liposomes dispersed in a borate-trehalose aqueous solution with sodium hyaluronate stored at 4 °C in the dark; *n* = three experiments.

**Table 4:** FLF pH, osmolarity, surface tension, and viscosity.

Storage Period (Weeks)	pH	Osmolarity (mOsm/L)	Surface Tension (mN/m)	Viscosity (mPa·s) *
0	7.47 ± 0.02	199 ± 2	36.5 ± 0.38	3.05 ± 0.02
2	7.44 ± 0.03	201 ± 1	37.4 ± 0.82	2.95 ± 0.08
4	7.46 ± 0.08	194 ± 1	37.7 ± 1.17	3.10 ± 0.03
8	7.45 ± 0.01	197.6 ± 1.7	35.0 ± 0.91	3.11 ± 0.02

FLF: Liposomes dispersed in a borate-trehalose aqueous solution with sodium hyaluronate stored at 4 °C in the dark; *n* = three experiments; * viscosity determined by applying the mathematic equation for Newtonian materials.

**Table 5:** Macroscopic evaluation of acute ocular tolerance of rabbits to instilled FLFs.

Sign/Symptoms	Grade *	Observation
Discomfort	0	No reaction
Corneal alterations	0	No alteration
Conjunctival alterations	0	No alteration
Discharge	0	No discharge
Lid alterations	0	No swelling

Drops (30 μL) were instilled every 30 min for 6 h. Observations were made prior to the first instillation and at 3, 6, and 24 h afterwards; * highest score recorded for the three evaluation periods.
